# Microencapsulation of *Saccharomyces cerevisiae* into Alginate Beads: A Focus on Functional Properties of Released Cells

**DOI:** 10.3390/foods9081051

**Published:** 2020-08-04

**Authors:** Antonio Bevilacqua, Daniela Campaniello, Barbara Speranza, Angela Racioppo, Clelia Altieri, Milena Sinigaglia, Maria Rosaria Corbo

**Affiliations:** Department of the Science of Agriculture, Food and Environment, University of Foggia, Via Napoli 25, 71122 Foggia, Italy; antonio.bevilacqua@unifg.it (A.B.); daniela.campaniello@unifg.it (D.C.); barbara.speranza@unifg.it (B.S.); angela.racioppo@unifg.it (A.R.); clelia.altieri@unifg.it (C.A.); milena.sinigaglia@unifg.it (M.S.)

**Keywords:** alginate, yeasts, functional properties, technological traits, release

## Abstract

Five yeast strains (four wild *Saccharomyces cerevisiae* strains and a collection strain-*S. cerevisiae* var. *boulardii*) were encapsulated in alginate beads. Encapsulation yield was at least 60% (100% for some strains) and yeasts survived in beads for 30 days at 4 °C, although the viability was strongly affected during storage at 25 °C (3 log reduction after 7 days). The kinetic of cell release was studied under static and dynamic conditions, but the results suggest that, after 48 h, beads contained a high number of yeasts. Thus, their use is advisable as re-usable carriers of starter cultures or as a vehicle of probiotics into the gut. Finally, some functional properties (biofilm formation, hydrophobicity, auto-aggregation, survival during the transit into the gut) were evaluated on yeasts released by beads to assess if microencapsulation could negatively affect these traits. The results showed that yeasts’ entrapment in beads did not affect probiotic properties.

## 1. Introduction

Microencapsulation is a successful microtechnology for food science and biotechnology to design immobilization systems, which protect active compounds (lipids, proteins, vitamins, enzymes, bacteria) that are otherwise subjected to rapid inactivation and/or degradation [1]. Many researchers use the terms immobilization and encapsulation as synonymous, despite their different meaning. Encapsulation means entrapment of an active ingredient in a shell by forming a continuous coating; on the other hand, immobilization is the process through the ingredient is linked to a matrix (also on the surface) [2].

In particular, “microencapsulation” was defined as “the technology of packaging solid, liquid and gaseous active ingredients in small capsules that release their content at controlled rates over prolonged periods of time” [2,3].

Microencapsulation systems find application in different sectors, from the pharmaceutical to the agri-food sector. In food biotechnology, they can be used to protect lactic acid bacteria in foods or in the gastrointestinal tract and may contribute to the development of new functional foods [4]. Microorganisms, in fact, could experience a strong viability loss as a result of harsh conditions (stomach, intestine, acidic environments in fermented foods, variations of temperatures, humidity, oxygen, and mechanical forces) [5]. Therefore, a barrier could protect them from the stresses encountered during food preparation processes and storage, as well as in the gut [6]. Microencapsulation, in fact, protects cells from mild heat treatments, adverse conditions characteristic of the gastrointestinal tract or, during storage, refrigeration [7,8,9].

Probiotics could be loaded in capsules through coacervation, emulsion, extrusion, spray-drying, and gel-particle technologies (including spray-chilling) [4]. One of the most common technique is the extrusion in a gel matrix of alginate. Alginate is widely used because it is a low-cost compound, it is biocompatible, non-toxic, and extracted from natural sources [6].

Alginate beads can entrap probiotics and starter bacteria [10,11,12,13]. However, few papers are available on yeasts, despite the fact that yeast entrapment and release over time could be very useful for different purposes, e.g., in mixed fermentations for a better control of microbial interactions, for a delayed release in the gut to extend bioactivity over time and promote the synthesis of useful compounds [14,15,16,17]. In a recent study, *Saccharomyces boulardii* was microencapsulated in alginate beads by emulsion and internal gelation, and yeast viability under in vitro and in vivo conditions was evaluated. The results showed that microencapsulation protected the yeast in adverse conditions [14]. Gallo et al. [15] carried out the microencapsulation of *S. boulardii* in sodium alginate microcapsules, and studied encapsulation yield (EY), cell viability throughout storage, and cell release kinetics. The results confirmed that microencapsulation assured yeast survival as well as its controlled release.

In another study, the encapsulation of *S. boulardii* was used to design new functional food like cheeses and yogurts [16] like a functional freeze-dried yogurt [17]. The microencapsulation increased the viability of yeast and extended the full benefits of the product compared to product supplemented with free or non-encapsulated yeast.

Few data are available on the effect of microencapsulation on the functional properties of bacteria [18] and to the best of our knowledge this topic is unexplored for yeasts.

Therefore, the main aim of this paper was to study an alginate-encapsulation system for foodborne yeasts to address the following topic: (a) the yield of the system; (b) the release kinetic of yeasts from beads; (c) the ability of beads to protect yeasts throughout gastrointestinal tract; (d) to study the effect of microencapsulation on some functional properties linked to surface properties of yeasts and related to their ability to adhere to gut mucosa (hydrophobicity, biofilm formation, auto-aggregation).

## 2. Materials and Methods

### 2.1. Microorganisms

Five microorganisms were used in this research: (i) *S. cerevisiae* var. *boulardii* ATCC MYA-796 purchased from American Type Culture Collection; (ii) *S. cerevisiae* W13, isolated from Uva di Troia, a grape variety of Southern Italy and able to remove ochratoxin A [19]; (iii) and three *S. cerevisiae* strains (2-4-17), isolated from Altamura sourdough, studied for their probiotic and technological properties [20]. The strains were stored at 4 °C on YPG agar (Bacteriological Peptone, 20 g/L; Glucose, 20 g/L; Yeast Extract, 10 g/L; Agar Technical, 12 g/L), and grown in YPG broth at 30 °C for 24 h.

### 2.2. Microencapsulation into Alginate Beads

The strains were inoculated in 500 mL-YPG broth and incubated at 30 °C for 48 h. Broth (20 mL) was centrifuged (8000 rpm for 15 min) to harvest cells and suspend them in 20 mL sterile water (cell suspension). A quantity of 0.4 g of Na-alginate (2%) (Fluka, Milan, Italy) was added to this suspension and mixed for 2 min, until a gel was formed. Gel drops were dipped through a sterile 10-mL-syringe in a sterile 0.5% CaCl_2_ solution (J.T. Baker, Milan, Italy). Beads were produced under sterile conditions.

Encapsulation yield was evaluated as reported by Corbo et al. [21], and Chavarri et al. [22]. Five grams of beads were diluted with 45 mL of sodium citrate (0.1 M) and homogenized through a laboratory blender. Homogenates and cell suspension before alginate addition were serially diluted in a saline solution (0.9% NaCl) and plated on YPG agar, incubated at 30 °C for 48 h. The analyses were performed on two independent batches (that is two different productions of beads on two different times); for each batch, the experiments were performed in duplicate over two different samples. The encapsulation yield (EY) was evaluated as follows:

EY = (N_bead_/N_suspension_)∗100 (1)

where N_bead_ (cfu/g) and N_suspension_ (cfu/g) are the viable counts in the beads after the immobilization and in cell suspension before the addition of alginate, respectively.

### 2.3. Yeast Viability During Storage

Beads were produced as reported above, and stored at 25 °C for 7 days or at 4 °C for 40 days. Yeast viable count was evaluated as reported above.

### 2.4. Release of Yeasts from Beads

The kinetic of cell release from alginate beads was evaluated immediately after bead production and on the beads stored under refrigerated conditions for 40 days. A quantity of 5 g of capsules was put in saline solution (50 mL). For each strain two experiments were performed: static or dynamic conditions (orbital shaker at 110 rpm). The samples were stored at room temperature and the conditioning medium was analyzed after 6, 24 and 48 h by plating. After 48 h, the number of cells not released by beads was analyzed as described above.

### 2.5. Hydrophobicity

The hydrophobicity was evaluated as reported by Bautista-Gallego et al. [23] on yeasts released by beads and on free cells. The strains were centrifuged (4000 rpm for 10 min), twice in PBS (Phosphate Buffer saline, Sigma-Aldrich, Milan, Italy), and finally diluted in 10 mL of 0.1 M KNO_3_ (C. Erba, Milan, Italy); the absorbance of this last suspension at 600 nm was coded as A_0_.

Three ml of xylene were added; after 10 min at room temperature (static conditions) the samples were mixed and left again at room temperature for 3 h to read the absorbance of the aqueous phase after 20 min, 1 h, 2 h, and 3 h (A_1_). Hydrophobicity (H%) was evaluated as follows:

H = [1−(A_1_/A_0_)]∗100 (2)

### 2.6. Auto-Aggregation

This assay was performed on yeasts released by beads and on free cells. Yeasts released in the conditioning solution were harvested by centrifugation, washed twice with PBS (phosphate saline buffer, 9 g/L di NaCl e 0.30 g/L Na_2_HPO_4_·2 H_2_O, Sigma-Aldrich). The resulting solution (5 mL) was left at room temperature (25 °C) to evaluate the absorbance at 600 nm of the upper suspension every hour. The experiments were performed at least in duplicate. The formula for auto-aggregation reads as follows [23]:

A = [1−(A_t_/A_0_)]∗100 (3)

where A_t_ and A_0_ are the absorbance at the time t and the initial value, respectively.

### 2.7. Simulated Gastrointestinal Conditions

Tolerance to simulated gastrointestinal conditions was evaluated using the method reported by Petruzzi et al. [19]. Three solutions were prepared and sterilized through filtration as follows:

Salivary conditions (SS): solution at pH 6.5, supplemented with 0.22 g/L CaCl_2_ (C. Erba, Milan, Italy), 6.5 g/L NaCl (C. Erba), 2.2 g/L KCl (J.T. Baker, Milan, Italy), 1.2 g/L NaHCO_3_ (Sigma-Aldrich), 100 mg/L lysozyme (Sigma-Aldrich) [24].

Gastric conditions (SGJ): saline solution (0.9% NaCl, pH 2.0) with 3 g/L pepsin (porcine gastric mucosal, Sigma-Aldrich) [24].

Intestinal conditions (SIF): 1 g/l pancreatin (porcine pancreas, Sigma-Aldrich), 3 g/L of bile extract (bile extract porcine, Sigma-Aldrich), 6.5 g/L NaCl, 0.835 g/L KCl, 0.22 g/L CaCl_2_, 1.386 g/L NaHCO_3_, pH 8 [24].

The assay was performed as follows:

1. Nine different sterile tubes, containing 45 mL of SS and 5 g of beads, were prepared and incubated at 37 °C for 5 min. Then, viable count was evaluated on beads and in SS from 3 tubes.
2. The beads were recovered from the remaining 6 tubes, suspended in SGJ (45 mL) and incubated at 37 °C for 120 min under agitation (200 rpm) [25,26]. Then, viable count was evaluated on beads and in SGJ from 3 tubes.
3. Beads were recovered from the remaining 3 tubes, suspended in SIF (45 mL) and incubated at 37 °C for 240 min, under agitation (200 rpm) [25,26]. Viable count was evaluated on beads and in SIF.

A second test was also performed, by suspending beads only in SS (salivary), SGJ (gastric) or SIF (intestinal).

The same test was also carried out on free cells. After each step, cells were harvested by centrifugation (4000 rpm-10 min). Analyses were performed on two different batches. Viable count was determined before and after each phase.

### 2.8. Biofilm Formation

The experiment was performed on both free cells and cells released from beads. Glass slides (25.4 mm × 76.2 mm) were used as surfaces. Before each experiment, slides were treated with acetone, 3.5% sodium hypochlorite (*v*/*v*) at 75 °C for 5 min, and 7.0 g/L phosphoric acid solution for 5 min. Then, the slides were rinsed in distilled water, air dried and autoclaved at 121 °C for 15 min [27].

A glass slide was put in a tube containing 40 mL of YPG; the broth was inoculated to 5 log cfu/mL with cells released from beads. Samples inoculated with free cells were used as positive controls.

A second experiment was performed by putting in the same sample beads and glass slide. For this second experiment, *S. cerevisiae* var. *boulardii* ATCC MYA-796 was used as a model organism.

The samples were prepared as follows:

1. 40 mL YPG broth+ beads (5 g)
2. 40 mL saline solution + beads (5 g)
3. 40 mL YPG broth + free cells (5 log cfu/mL)
4. 40 mL saline solution + free cells (5 log cfu/mL)

The samples were incubated at 25 °C for 14 days. The populations in planktonic and sessile states were periodically determined by a standard plate count on YPG Agar. Slides were removed from the medium, gently washed with sterile distilled water to remove the unattached cells and placed into a test-tube containing 45 mL of sterile saline solution and sonicated at 20% power “Vibra Cell” for 3 min [27].

### 2.9. Statistic

The experiments were performed on two or three independent batches; the results were analyzed through a t-student’s test (paired comparison, *p* < 0.05) or one-way ANOVA using the Tukey’s test as the *post-hoc* test (multiple comparison). Statistic was made through the software Statistica for Windows ver. 12.0 (Stasoft, Tulsa, OK, USA). All data in figures and tables are shown as mean values ± standard deviation.

## 3. Results and Discussion

The first parameter assayed in this research was EY (encapsulation yield), that is the percentage of cells entrapped into alginate, as reported elsewhere [15,21,28] (Table 1). EY was between 87.70 and 108% for the strains 4, 17 and SB (*S. cerevisiae* var. *boulardii*), while the strains 2 and W13 showed values of 54.07 and 62.78%, respectively. Some preliminary findings suggest that this lower EY was probably due to a reversible stress on cells, generally known as “crowding” and found for these strains when they are at high levels. A first experiment performed on entrapped yeasts suggest the idea that some yeasts could experience a transient viable but not culturable cells, probably responsible for a lower EY; however, EY was calculated using the exponential values of the cell counts, i.e., EY > 50% means a difference in cell concentration between the suspension and the beads of 0.5 log cfu/mL or lower.

Table 2 shows the viability of yeasts in beads during storage at 4 °C for 30 days, and 25 °C for 7 days. The initial concentration of yeasts in the beads at 4 °C was 7.07–7.81 log cfu/g and during the storage there were not significant changes. These data confirmed the results of Gallo et al. [15] who reported the survival of *S. boulardii* into the same kind of beads for 90 days. Moreover, the goodness of the technique was also confirmed by Suvarna et al. [29], who assessed the viability of yeasts at 4 °C for 30 days, testing different kinds of gel matrix for encapsulation (sodium alginate, chitosan coated sodium alginate, sodium alginate-gelatinized starch). On the other hand, the viability of yeasts in beads at 25 °C for 7 days (“stress test”), showed a significant reduction of the viable count.

The viability of encapsulated cells is influenced by the type and the concentration of the surrounding polymer, particle size, initial cell numbers and strains. Some authors proposed alginate as a good polymer for microencapsulation due to some benefits [30,31]: it is a nontoxic, biodegradable and biocompatible polymer [32] and shows a good stability of matrix under mild conditions at ambient temperature [33]. Moreover, microencapsulation can be considered a promising method for the protection of bacteria or yeasts sensitive to high temperature [14,34].

Several studies have shown that microencapsulation in alginate microparticles also improve the survival of probiotic bacteria [21,35,36,37].

The results of this research confirm the suitability of this approach due to the prolonged survival of cells into beads at least at 4 °C. Nowadays, there are several industries focusing on probiotic market with an increasing interest, and microencapsulation can be an efficient method of extending the shelf life of probiotic food products. There are already several foods on the market containing encapsulated probiotic cells, such as chocolate, yogurt and ice cream [4].

The second phase for the optimization of the method was the evaluation of the kinetics of cell release from beads. This is an important parameter if the beads are produced as a carrier to release microorganisms in specific environments. Indeed, an advantage of the microencapsulation system is the controlled release of entrapped cells [38,39,40].

Therefore, the kinetic of yeast release was studied as a function of different variables: agitation of the conditioning medium (static and dynamic conditions) and age of beads (used immediately after gelling or studied after a preliminary refrigerated storage).

Table 3 shows the kinetic of cell release of yeasts in fresh beads. Beads released cells after 6 h (4.26 and 4.17 log cfu/mL for the strains SB and W13, ca. 3 log cfu/mL for the strains 2, 4, and 17); under static conditions, beads with strains 17 and 4 did not show a kinetic of cell release, while the strains SB, W13 and 2 released ca. 3 log cfu/mL, although at different time intervals (after 6 h for the strains SB and W13 and after 24 h the strain 2). The storage at 4 °C for 40 days did not affect this trend (data not shown).

**Figure 1:**
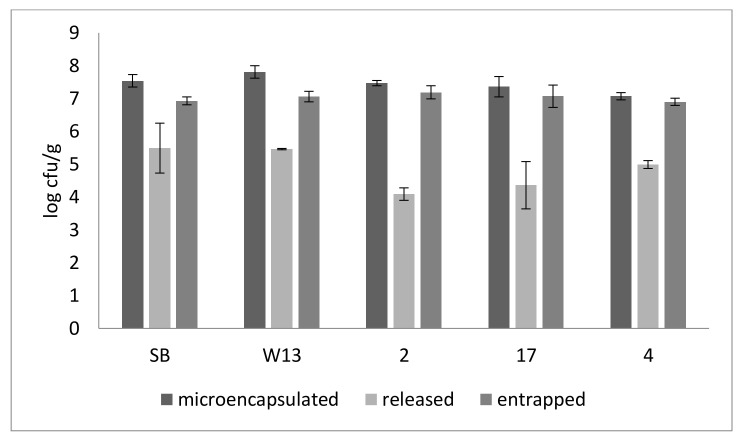
Cell concentration of yeasts beads, released yeasts, and entrapped in the beads (log cfu/g), after 48 h. SB, W13, 2, 17, 4, strains; SB: *S. cerevisiae* var. *boulardii.* The results correspond to fresh beads (not stored at 4 °C).

By combining the results of EY and the kinetic of cell release from beads, the effective amount of cells released after 48 h was evaluated; the amount of cells effectively released in the conditioning medium was 4.09–5.49 log cfu/g (for the strains 1 and SB, respectively), that is 1% or less of the total amount of cells. Therefore, after 48 h, beads contained a high number of cells (6.90 log cfu/g for the strain SB and 7.19 log cfu/g for the strain 2) (Figure 1).

For lactic acid bacteria, many authors assumed that initially the capsules released cells contained into the outer layers; then, the cells of the inner layers were released, following their migration to the superficial layers [21,28,41]. This hypothesis was also reported by Gallo et al. [15] for yeasts. However, an analysis of the gel structure should be done to verify these hypotheses.

These results suggest that alginate beads are suitable carriers to release cells in the gut, where alginate is generally disrupted and all cells can be released; on the other hand, the release in some media is lower and this trait suggests the possibility of using beads as re-usable carriers to start a fermentation for 7–10 different batches [15].

Delayed kinetic of cell release could be exploited in alcoholic fermentation processes, since the cell growth in the beads contribute to increase the final ethanol concentration. Moreover, microencapsulated yeasts could be used in a continuous fermentation process due to several advantages such as the ease of cell separation from the medium, a cost reduction due to the reuse of cells in subsequent reaction cycles and a reduced possibility of contamination, as reported by different authors [42,43].

After assessing the optimization of method, the effect of microencapsulation was studied on some selected functional properties of yeasts (hydrophobicity, auto-aggregation, biofilm formation, survival). Hydrophobicity, auto-aggregation, and biofilm formation are indirect tools to assess the ability of microorganisms to adhere to gut mucosa [44,45,46,47].

**Figure 2:**
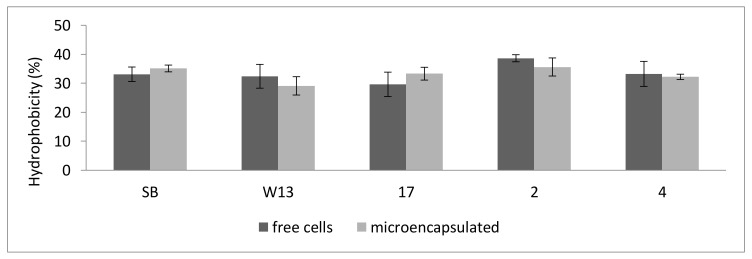

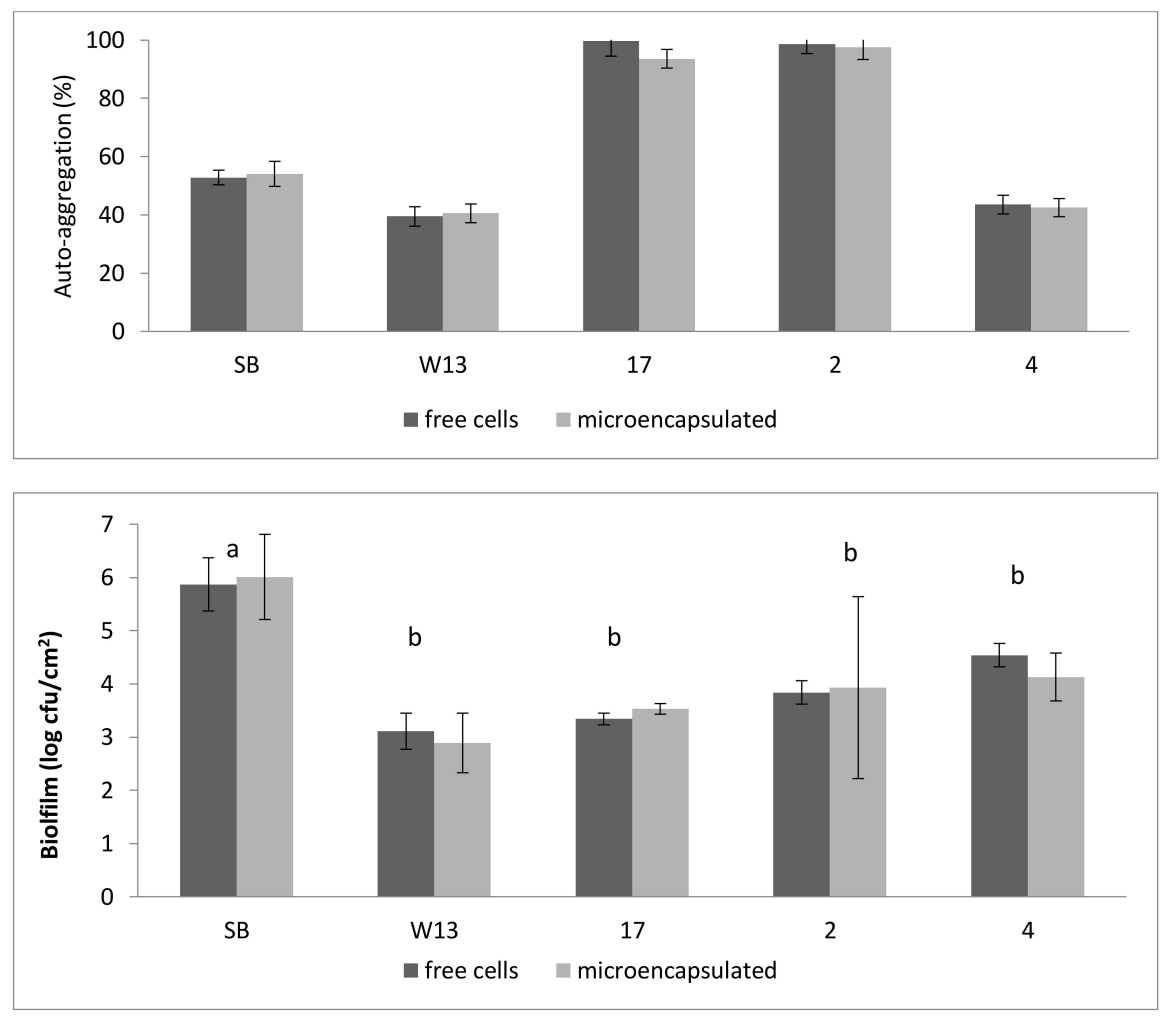
Effect of microencapsulation on some functional properties of yeasts: hydrophobicity (%); auto-aggregation after 2 h (%); biofilm formation after 5 days (log cfu/cm^2^). The properties were evaluated on cells released by beads. The letters indicate significant differences (one-way ANOVA and Tukey’s test, *p* < 0.05). SB, W13, 2, 17, 4, strains; SB: *S. cerevisiae* var. *boulardii.*

Figure 2 shows the effects of microencapsulation on hydrophobicity, auto-aggregation and biofilm formation for the cells released from beads. These properties were never affected by microencapsulation. Concerning biofilm formation, after 5 days, the strain SB shown the highest level of sessile cells (5.87 log cfu/cm^2^), followed by strains 2, 4, 17, and W13.

All these experiments were carried on cells released by beads and harvested by centrifugation; a second test was performed to simulate a condition with beads directly in contact with mucosa. Therefore, beads were put in tubes along with glass slides, and biofilm formation was evaluated without a preliminary step of cell harvesting; the experiment was done only with the strains SB and 17, in two media: a laboratory substrate and a minimal medium (Table 4).

Biofilm formation was only found in the lab medium for both SB and strain 17; however, the trend was different for the two yeasts. Biofilm was found after 8 h for both free and entrapped cells for strain 17, without differences in the concentration of sessile cells. On the other hand, free cells of SB produced a biofilm after 5 h (2.45 log cfu/cm^2^) and then the concentration increased after 8 h to ca. 4 log cfu/cm^2^. Cells released from beads produced a biofilm after 8 h at 3 log cfu/cm^2^.

Similar results were found by Bevilacqua et al. [18], for *Lacticaseibacillus casei* and *Bifidobacterium bifidum*. They showed that microencapsulation did not modify the hydrophobicity of cells.

The breakpoint for a is 10^6^–10^7^ cfu per g, during the shelf-life [48]. Thus, processors should fulfill this requirement and guarantee the viability of probiotic until the time of consumption; thus, the strain remains viable and reaches the colon where it should proliferate, exerting its beneficial probiotic effects [16,17,49].

In this research, the ability of beads to protect yeasts throughout gastrointestinal tract was evaluated using the method reported by Petruzzi et al. [19]. In the first step, each phase was tested separately (salivary condition, gastric conditions, intestinal condition), and in the second step a sequential protocol was evaluated. Free cells were used as a control. Table 5 shows the viability of free cells, and yeasts in beads in a simulated gastrointestinal tract. As expected, none of the tested control strains showed statistically significant differences between the concentration before and after exposure to each phase (*p* > 0.05). Similar results were obtained in the sequential protocol. After checking the intrinsic resistance of yeasts to gastrointestinal conditions, we studied the effect of microencapsulation to check whether beads could be able to protect cells. As regards phases tested alone, neither the salivary nor the gastric phase determined a significant reduction of the concentration of microencapsulated cells. However, after exposure to the intestinal phase, strain 2 showed a significant reduction in cell concentration (by 7.40 to 5.76 log cfu/g), thus suggesting a weakening of the microencapsulated strain. Based upon this result, strain 2 was not subjected to the sequential protocol, as it was considered unsuitable.

The other strains showed similar performances as free or entrapped cells when exposed to the sequential protocol. The survival of yeasts after the exposure to gastrointestinal conditions is a strain characteristic and some entrapment systems could improve it. Some authors reported that microencapsulation increased the survival of the probiotic micro-organisms in simulated gastric juice [41,50,51], whilst others did not observe any effect in gastric and bile juice [36] or found a slight effect (0.5 log cfu/mL improvement in Pinpimai et al. [52]).

The data of this research suggest that yeasts loaded in alginate microspheres survived when exposed to simulated gastrointestinal conditions, and confirmed the results reported by Gallo et al. [15]. A similar gastro-resistance was reported by Suvarna et al. [29] and Qi et al. [14]. However, the main finding of this paper is the use of a low amount of alginate (2%) without adverse effect on the viability.

The amount of alginate in beads is linked to cell release from capsules (a higher amount means a reduced release of cells) as well as to strain resistance to some stress conditions (higher amounts confer a higher protection) [53,54], thus the choice of alginate amount is the result of a balance between protection and release. The results of this paper with an enhanced viability also for a low concentration of the polymer suggest that this kind of approach is suitable for yeasts, because it is possible to maximize or enhance their release without affecting their viability into the gut.

## 4. Conclusions

In conclusion, this study proposes a first structured approach to evaluate the effect of microencapsulation into alginate gels on the functional properties of yeasts. The results suggest that the confinement of yeasts in beads did not affect probiotic properties (hydrophobicity and biofilm formation), and was able to protect the cells into simulating gastrointestinal conditions. Finally, the kinetic of cell release suggest that, after 48 h, beads contain a high number of yeasts. Thus, their use is advisable as re-usable carriers of starter cultures or as vehicle of probiotics into the gut. Further investigations are required because, for one strain, a reduction of viability was found when cells were entrapped; this result needs to be confirmed to assess if some strains could experience a stress when confined in a restricted space.

## Figures and Tables

**Table 1:** Encapsulation yield of yeasts in beads (%). The letters indicate the significant differences (one-way ANOVA and Tukey’s test, *p* < 0.05). SB: *S. cerevisiae* var. *boulardii.*

Strains	EY (%)
SB	93.75 ± 0.36d
W13	62.78 ± 0.34b
17	108.94 ± 0.32e
2	54.07 ± 0.14a
4	87.80 ± 0.61c

**Table 2:** Viability of yeasts in beads (log cfu/g) during storage at 4 °C for 30 days, and 25 °C for 7 days. For each strain, the letters indicate significant differences (one-way ANOVA and Tukey’s test, *p* < 0.05). SB: *S. cerevisiae* var. *boulardii*: * days.

	Strains				
Days at 4 °C	2	4	17	W13	SB
0	7.54 ± 0.19a	7.81 ± 0.19a	7.47 ± 0.08a	7.36 ± 0.32a	7.07 ± 0.11a
15	7.21 ± 0.19a	7.53 ± 0.20a	7.07 ± 0.40a	7.42 ± 0.08a	7.13 ± 0.29a
30	7.33 ± 0.23a	7.24 ± 0.17a	7.70 ± 0.16a	7.50 ± 0.18a	7.02 ± 0.32a
**Days at 25 °C**					
7	4.35 ± 0.23b	4.11 ± 0.35b	4.53 ± 0.23b	3.99 ± 0.11b	4.01 ± 0.22b

**Table 3:** Kinetic of cells release of yeasts in alginate beads (log cfu/g), under static or dynamic conditions. For each yeast, the letters indicate significant differences (one-way ANOVA and Tukey’s test, *p* < 0.05). SB: *S. cerevisiae* var. *boulardii.* The results correspond to fresh beads (not stored at 4 °C).

Strains					
Time (h)	SB	W13	2	17	4
Dynamic Conditions					
**0**	- *	-	-	-	-
**6**	4.26 ± 0.68a,b	4.17 ± 0.08a	2.97 ± 0.06b	2.53 ± 0.08c	3.17 ± 0.13b
**24**	4.49 ± 0.76a	4.32 ± 0.05a	3.06 ± 0.09b	2.62 ± 0.10c	3.37 ± 0.29b
**48**	3.90 ± 0.18a	4.46 ± 0.02b	3.09 ± 0.19c	3.36 ± 0.72d	3.99 ± 0.12a
**Static Conditions**					
**0**	-	-	-	-	-
**6**	3.30 ± 0.09	3.06 ± 0.00	-	-	-
**24**	3.28 ± 0.03	3.04 ± 0.02	3.18 ± 1.42	-	-
**48**	-	3.20 ± 0.04	3.03 ± 0.23	-	-

* The symbol “^_”^ means “below the detection limit”

**Table 4:** Biofilm formation (log cfu/cm^2^) of S. *cerevisiae* var *boulardii* and *S. cerevisiae* 17: beads were put in the same solution of glass slides. Optimal medium (Y) and saline solution (S). B, cells in beads; F, free cells. The letters indicate significant differences in a column (one-way ANOVA and Tukey’s test, *p* < 0.05). SB: *S. cerevisiae* var. *boulardii.*

SB				
Time (h)	YF	YB	SF	SB
**0**	-P	-	-	-
**5**	-	2.45 ± 0.12d	-	-
**8**	3.24 ± 0.01c	4.46 ± 0.21a	-	-
**14**	3.17 ± 0.01c	4.25 ± 0.02b	-	-
**21**	3.06 ± 0.12c	4.29 ± 0.02b	-	-
17				
**0**	-	-	-	-
**5**	-	-	-	-
**8**	3.07 ± 0.05c	2.95 ± 0.02c,d	-	-
**14**	3.13 ± 0.02c	3.21 ± 0.04c	-	-
**21**	2.86 ± 0.14c,d	3.28 ± 0.01c	-	

* The symbol “-“ means below the detection limit.

**Table 5:** Viability of free cells, and yeasts in beads (log cfu/mL) during simulated gastrointestinal tract (GIT). Each phase was tested separately before and after simulated gastrointestinal tract (GIT), and sequentially. F, free cells; B, microencapsulated. SB: *S. cerevisiae* var. *boulardii.*

Separate Phases												
Strains	Salivary Conditions				Gastric Conditions				Intestinal Conditions			
	F		B		F		B		F		B	
	Before	After	Before	After	Before	After	Before	After	Before	After	Before	After
**2**	7.65 ± 0.21	7.61 ± 0.25	7.40 ± 0.11	7.22 ± 0.17	7.15 ± 0.62	7.18 ± 0.61	7.40 ± 0.11	6.74 ± 0.18	7.15 ± 0.62	6.98 ± 0.44	7.40 ± 0.11	5.76 ± 0.22
**4**	7.52 ± 0.14	7.22 ± 0.08	7.40 ± 0.23	7.05 ± 0.24	7.2 ± 0.11	6.90 ± 0.53	7.44 ± 0.33	7.01 ± 0.17	7.23 ± 0.15	7.07 ± 0.02	7.44 ± 0.23	6.97 ± 0.25
**17**	7.35 ± 0.11	7.11 ± 0.35	7.40 ± 0.20	7.68 ± 0.06	7.35 ± 0.62	7.38 ± 0.21	7.20 ± 0.20	7.78 ± 0.19	7.25 ± 0.62	6.97 ± 0.44	7.20 ± 0.20	7.20 ± 0.27
**W13**	7.65 ± 0.01	7.60 ± 0.06	7.40 ± 0.11	7.40 ± 0.18	7.27 ± 0.22	7.29 ± 0.05	7.40 ± 0.11	7.42 ± 0.22	7.4 ± 0.03	7.30 ± 0.1	7.40 ± 0.11	7.22 ± 0.08
**SB**	7.42 ± 0.10	7.4 ± 0.11	7.40 ± 0.11	7.60 ± 0.07	7.06 ± 0.03	7.30 ± 0.08	7.40 ± 0.11	7.74 ± 0.25	7.35 ± 0.25	7.25 ± 0.15	7.40 ± 0.11	6.76 ± 0.28
		**Sequential Protocol**										
		**Strains**	**Before**		**Salivary Conditions**		**Gastric Conditions**		**Intestinal Conditions**			
			**F**	**B**	**F**	**B**	**F**	**B**	**F**	**B**		
		**4**	7.52 ± 0.15	7.44 ± 0.30	7.22 ± 0.20	7.05 ± 0.24	7.29 ± 0.24	7.35 ± 0.09	7.21 ± 0.13	6.72 ± 0.60		
		**17**	7.40 ± 0.10	7.20 ± 0.20	7.30 ± 0.15	7.38 ± 0.30	7.35 ± 0.13	6.82 ± 0.60	7.41 ± 0.17	7.20 ± 0.27		
		**W13**	7.66 ± 0.01	7.40 ± 0.21	7.60 ± 0.05	7.40 ± 0.20	7.30 ± 0.29	7.42 ± 0.11	7.45 ± 0.03	6.62 ± 0.15		
		**SB**	7.42 ± 0.10	7.30 ± 0.19	7.66 ± 0.06	7.35 ± 0.33	7.48 ± 0.25	7.29 ± 0.23	7.39 ± 0.14	7.31 ± 0.28		
